# Spontaneous Remission of Acquired Generalized Lipodystrophy Presenting in the Postpartum Period

**DOI:** 10.1210/jcemcr/luae009

**Published:** 2024-02-02

**Authors:** Ranvir Bhatia, Prathyusha Chennupathi, Elliot D Rosenstein, Sonoo Advani

**Affiliations:** Perelman School of Medicine, University of Pennsylvania, Philadelphia, PA 19104, USA; Division of Rheumatology, Overlook Medical Center, Atlantic Health System, Summit, NJ 07901, USA; Division of Rheumatology, Overlook Medical Center, Atlantic Health System, Summit, NJ 07901, USA; Atlantic Medical Group, Atlantic Health System, Morristown, NJ 07960, USA; Atlantic Medical Group, Atlantic Health System, Morristown, NJ 07960, USA; Division of Endocrinology, Overlook Medical Center, Atlantic Health System, Summit, NJ 07901, USA

**Keywords:** acquired generalized lipodystrophy, insulin resistance, anti-Perilipin-1, u-500 insulin, thiazolidinediones

## Abstract

Acquired generalized lipodystrophy (AGL) is a rare condition characterized by the diffuse loss of adipose tissue resulting in hyperglycemia, severe insulin resistance, and sequelae of metabolic disease. Here, we report the case of a 32-year-old woman who developed uncontrolled hyperglycemia and significant weight loss within 2 months postpartum. Upon endocrine evaluation, she was found to have generalized loss of adiposity, hypoleptinemia, and persistent hyperglycemia despite aggressive insulin administration. Glycemic response was obtained with U-500 intramuscular insulin, pioglitazone, and metformin-sitagliptin. At 14 months postpartum, the patient achieved spontaneous remission with normoglycemia off medication and restoration of adipose tissue deposition. Autoimmune workup revealed positive antinuclear antibodies (ANA) and anti-U1-ribonucleoprotein (anti-U1-RNP) titers, suggestive of an autoimmune etiology to her condition. This case of AGL represents the first reported case of spontaneous remission and the first to develop in the postpartum period.

## Introduction

With just over 100 reported cases to date, acquired generalized lipodystrophy (AGL) is a rare condition characterized by the generalized loss of adipose tissue resulting in severe insulin resistance, hyperglycemia, hypertriglyceridemia, and hepatic steatosis [1]. It is estimated that 25% of cases of AGL are characterized by panniculitis at onset, 25% are associated with autoimmune disease, and the remainder are idiopathic in etiology [2]. While there is a low prevalence of metabolic derangements in the panniculitis subtype, diabetes and hypertriglyceridemia have each been reported to occur in 80% to 90% of patients with autoimmune or idiopathic AGL. It has been recently reported that 37% of patients with AGL produce autoantibodies to perilipin-1 (anti-PLIN1), a protein mutated in a subtype of familial partial lipodystrophy [3]. This disease subset is enriched in clinical features of autoimmunity. PLIN1, one of the most abundant proteins in mature white adipocytes, is responsible for the regulation of lipid incorporation and release. In functional studies, incubation with anti-PLIN1 autoantibodies increased basal lipolysis in cultured preadipocytes, revealing a potential causal relationship [1]. Nonetheless, much remains to be ascertained in terms of pathophysiology, disease biomarkers, and optimal treatment of the condition. The case we present here is remarkable in 2 ways: it is both the first reported case of AGL to undergo spontaneous remission and the first to develop in the postpartum period.

## Case Presentation

A 32-year-old G4P2022 woman presented to the endocrinology clinic in June 2022 for evaluation of recently diagnosed type 2 diabetes. She reported that 2 months after the delivery of her second child in August 2021, she noticed more than 34 kg of weight loss, fatigue, and increased hunger. Her course included vomiting, polyuria, polydipsia, and blurred vision. She was noted to have marked hyperglycemia (blood glucose > 27.75 mmol/L) (500 mg/dL) (reference range [RR], 3.89-5.49 mmol/L) requiring admission for management of diabetic ketoacidosis (DKA). At the time of presentation to clinic, her regimen consisted of glargine 60 units twice daily, lispro U-100 40 units 3 times daily with meals, and sitagliptin-metformin extended release 100 to 2000 mg daily.

The patient’s obstetric history was notable for one induced abortion in 2013, one spontaneous first-trimester abortion in 2020, one full-term, uncomplicated spontaneous vaginal delivery, and one full-term cesarean delivery due to arrest of descent. There was no history of gestational diabetes or pregnancy-induced hypertension in any of her prior pregnancies. During her most recent pregnancy, a 1-hour oral glucose tolerance test obtained at 28 weeks revealed blood glucose of 7.71 mmol/L (139 mg/dL) (RR, < 7.21 mmol/L), indicative of abnormal glucose tolerance. This pregnancy was complicated by a low PAPP-A value on first-trimester screening and mild gestational thrombocytopenia. The patient denied a history of any medical conditions prior to this pregnancy. Family history was notable for a sister with Crohn disease.

## Diagnostic Assessment

**Figure 1.**
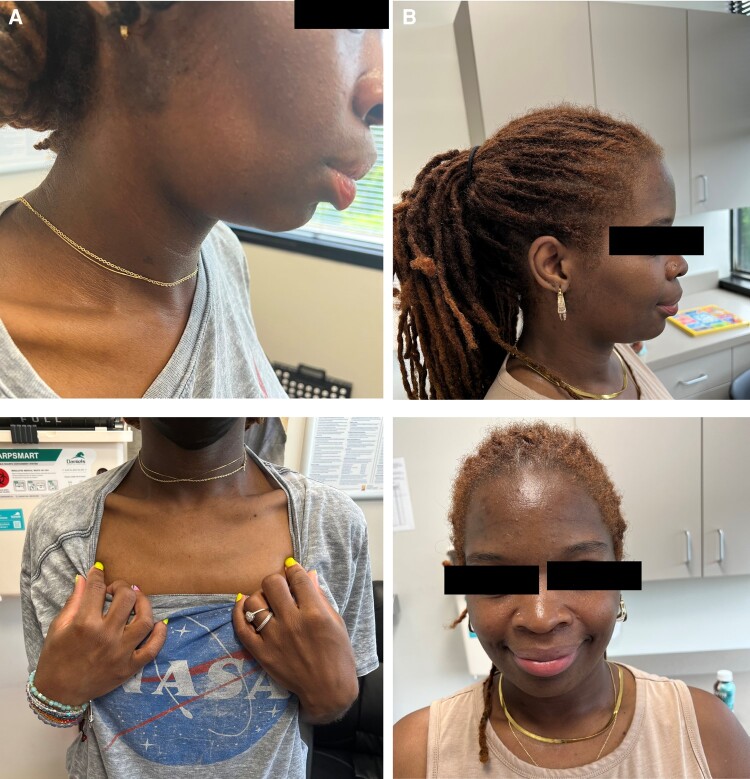
Physical examination of patient upon initial visit in June 2022 (A) and follow-up July 2023 (B).

Upon physical examination, the patient weighed 59.0 kg with a body mass index (BMI) of 23.0 kg/m^2^, 39.9 kg less than her postpartum discharge weight (Table 1). She was found to have a diffuse absence of adipose tissue on her face, chin, upper, and lower extremities (Fig. 1). Her thyroid was soft, diffuse, and nontender. No acanthosis nigricans or skin tags were observed. No erythematous, nodular, or ulcerative lesions suggestive of panniculitis were identified. The remainder of her examination was normal, without lymphadenopathy, hepatosplenomegaly, joint swelling, or skin lesions.

**Table 1.** Metabolic parameters over disease course

Date	Pregnancy timeline	Weight	BMI	HbA1c	C-peptide (RR, 0.363-1.45 nmol/L)	Leptin
1/11/21	8 weeks per LMP	94.3 kg	36.8 kg/m^2^	—	—	—
8/14/21	Delivery at 38 weeks	98.9 kg	38.6 kg/m^2^	—	—	—
	Months Postpartum					
6/8/22	10	59.0 kg	23.0 kg/m^2^	15.5% (146 mmol/mol)	9.27 ng/mL (3.07 nmol/L)	1.9 ng/mL (0.118 nmol/L) (RR 0.236-1.30 nmol/L)
7/20/22	11	62.1 kg	24.3 kg/m^2^	—	—	—
12/2/22	13	72.6 kg	29.3 kg/m^2^	12.7% (115 mmol/mol)	5.08 ng/mL (1.68 nmol/L)	—
1/9/23	14	80.7 kg	32.6 kg/m^2^	—	—	—
3/29/23	16	—	—	5.6% (38 mmol/mol)	3.29 ng/mL (1.09 nmol/L)	—
7/26/23	20	87.5 kg	35.3 kg/m^2^	5.2% (33 mmol/mol)	1.0 ng/mL (0.33 nmol/L)	65.7 ng/mL (4.08 nmol/L) (RR 1.37-7.52 nmol/L)

Abbreviations: BMI, body mass index; HbA1c, hemoglobin A1C; LMP, last menstrual period; RR, reference range.

Records from her initial hospitalization for DKA revealed hemoglobin A1C (HbA1c) > 14.0% (>130 mmol/mol) (RR, 4.0%-5.6% or 20.2-37.7 mmol/mol), C-peptide 0.29 nmol/L (0.88 ng/mL) (RR, 0.165-1.02 nmol/L), negative GAD65, ZNT8, IA-2, and insulin antibodies, persistent hyperglycemia with blood glucose readings > 16.5 mmol/L (300 seconds mg/dL) despite aggressive insulin administration (over 140 units daily), white blood cell count (WBC) 1.5 × 10^3 ^cells/µL (RR, 4.0-10.5 × 10^3 ^cells/µL), absolute neutrophil count (ANC) 0.5 × 10^3 ^cells/µL (RR, 2.19-7.53 × 10^3 ^cells/µL), hemoglobin 7.32 mmol/L (11.8 g/dL) (RR, 7.45-9.74 mmol/L), and platelet count (PLT) 82 × 10^3 ^cells/µL (RR, 140-450 × 10^3 ^cells/µL) (Table 2), total cholesterol 2.64 mmol/L (102 mg/dL) (RR, < 5.18 mmol/L), high-density lipoprotein 0.47 mmol/L (18 mg/dL) (> 1.27 mmol/L), low-density lipoprotein 1.76 mmol/L (68 mg/dL) (< 2.59 mmol/L), triglycerides 0.90 mmol/L (80 mg/dL) (< 1.7 mmol/L). On peripheral blood smear, atypical lymphocytes and giant platelets were observed. Peripheral blood flow cytometry, serum and urine protein electrophoresis, copper, HIV, viral hepatitis panel, lactate dehydrogenase, and haptoglobin were unremarkable. Antinuclear antibody (ANA) titer was > 1:1280 with a speckled pattern (RR, < 1:40), anti-Smith antibody > 8.0 antibody index (RR, < 1), anti-U1-RNP > 8.0 antibody index (RR, < 1); her anti-Sjögren syndrome A and B antibodies (SSA, SSB), rheumatoid factor (RF), angiotensin-converting enzyme (ACE), and anti-neutrophil cytoplasmic antibody (ANCA) panel were unremarkable.

**Table 2.** Hematologic parameters over disease course

Date	Months postpartum	WBC (RR, 4.0-10.5 × 10^3 ^cells/µL)	ANC (RR, 2.19-7.53 × 10^3 ^cells/µL),	Hgb (RR, 7.45-9.74 mmol/L)	PLT (RR, 140-450 × 103 cells/µL)
8/15/21	0	12.80 × 10^3 ^cells/µL	—	7.7 g/dL (4.78 mmol/L)	127 × 10^3 ^cells/µL
12/11/21	4	1.5 × 10^3 ^cells/µL	0.5 × 10^3 ^cells/µL	11.8 g/dL (7.32 mmol/L)	82 × 10^3 ^cells/µL
6/6/22	10	2.1 × 10^3 ^cells/µL	1.1 × 10^3 ^cells/µL	12.5 g/dL (7.76 mmol/L)	145 × 10^3 ^cells/µL
12/1/22	16	1.9 × 10^3 ^cells/µL	—	11.8 g/dL (7.32 mmol/L)	135 × 10^3 ^cells/µL
3/29/23	19	2.1 × 10^3 ^cells/µL	—	11.7 g/dL (7.26 mmol/L)	177 × 10^3 ^cells/µL
8/2/23	24	1.8 × 10^3 ^cells/µL	0.6 × 10^3 ^cells/µL	11.2 g/dL (6.95 mmol/L)	150 × 10^3 ^cells/µL

Abbreviations: ANC, absolute neutrophil count, Hgb, hemoglobin; PLT, platelet count; RR, reference range; WBC, white blood cell count.

Laboratory results from June 6, 2022, were notable for HbA1c > 15.5% (>146 mmol/mol), C-peptide 3.07 nmol/L (9.27 ng/mL), WBC 2.1 × 10^3 ^cells/µL, ANC 1100 cells/µL, PLT 145 × 10^3 ^cells/µL, thyrotropin (TSH) 2.08^ ^mIU/L (RR, 0.45-4.5), and normal liver function tests. A serum leptin level was 0.118 nmol/L (normal leptin range for BMI of 23 kg/m^2^, 0.236-1.30 nmol/L; equivalent to 1.9 ng/mL). Given her generalized absence of adipose tissue on examination, persistent hyperglycemia with significant insulin resistance, and low serum leptin level, the patient was diagnosed with AGL.

## Treatment

The patient was started on pioglitazone 45 mg daily, and her prandial insulin was changed to insulin-NPH-insulin-regular 70-30, 65 units twice a day before meals. One week later, in consideration of the patient’s significant paucity of subcutaneous fat, she was transitioned to an intramuscular insulin regimen of regular U-500 50 units twice a day. She was instructed to limit her total carbohydrate intake.

## Outcome and Follow-Up

On follow-up visits at 1 and 4 months, the patient had considerable improvement in her metabolic parameters. She reported symptomatic improvement in her fatigue and polyphagia.

At 4 months, notable deposition of adipose tissue was observed on her face, upper, and lower extremities. A lipid panel obtained during this visit was within normal limits: total cholesterol 2.77 mmol/L (107 mg/dL), high-density lipoprotein 0.98 mmol/L (38 mg/dL), low-density lipoprotein 1.43 mmol/L (56 mg/dL), and triglycerides 0.62 mmol/L (55 mg/dL). Her insulin was discontinued at this visit. On continuous glucose monitoring over the next month, the patient displayed fasting hypoglycemia with her oral medications and fasting sugars consistently between 3.9 mmol/L (70 mg/dL) and 5.0 mmol/L (90 mg/dL) without medication. Her oral medications were subsequently discontinued.

In March 2023, repeat laboratory tests showed a HbA1c 5.6% (38 mmol/mol), C-peptide 1.09 nmol/L (3.29 ng/mL), and unremarkable thyroid function panel. Repeat leptin level in July 2023 was 4.08 nmol/L (65.7 ng/mL) (normal leptin range for BMI of 35 kg/m^2^, 1.37-7.52 nmol/L). Repeat autoimmune workup revealed positive ANA 1:1280 in a speckled pattern, positive anti-U1-RNP antibody (163 AI), elevated erythrocyte sedimentation rate (ESR) (62 mm/hr; RR, 0-30), and serum protein electrophoresis with a polyclonal gamma globulin elevation (2.4 g/dL; RR, 0.4-1.8). Other than mild chronic fatigue, the patient offered no symptomatology of active systemic autoimmune disease and had an unremarkable examination. Given her positive autoimmune serologies from both her initial hospitalization and repeat evaluation, leukopenia, and polyclonal gammopathy, it was felt that her AGL was most likely autoimmune in nature. No serum was available from her initial hospitalization to assess for the presence of anti-PLIN1 antibodies. After a comprehensive hematologic evaluation, including repeat peripheral blood flow cytometry, repeat lactate dehydrogenase, Duffy antigen phenotyping, and myeloid next-generation sequencing, which were all unremarkable, her leukopenia and neutropenia were also felt to be autoimmune in nature. An abdominal ultrasound obtained on thrombocytopenia workup revealed no hepatosplenomegaly and no signs of hepatic steatosis. She will follow up with rheumatology for continued monitoring for signs of systemic autoimmune disease.

## Discussion

Within 2 months postpartum, our patient developed sudden onset weight loss with severe uncontrolled hyperglycemia. On endocrine evaluation, she required U-500 intramuscular insulin, pioglitazone, and metformin-sitagliptin for glycemic response. At 14 months postpartum, she achieved spontaneous remission with normoglycemia off medication and had restoration of adipose tissue deposition. At 21 months postpartum, she continues to remain in remission with an HbA1c of 5.2% (33 mmol/mol) off medication.

Notably, we felt that the patient’s low-normal serum C-peptide during her initial hospitalization was likely due to acute beta cell dysfunction in the setting of DKA. During her subsequent clinic visit 6 months later, her C-peptide was more than 3 times the upper limit of normal, consistent with the severely insulin resistant phenotype of AGL.

AGL is diagnosed clinically based on history, physical examination, and metabolic parameters [4]. While there currently exist no established diagnostic criteria, Misra et al have proposed diagnostic criteria based on 79 cases of AGL [2]. The essential criterion involves generalized loss of adiposity manifesting after birth. Supportive criteria include clinical and laboratory features of lipodystrophy, insulin resistance, and hypoleptinemia.

The patient’s high titer ANA with positive anti-U1-RNP antibody on repeated testing, persistent leukopenia, and polyclonal gammopathy support an autoimmune diathesis. Despite her serologic profile, she had no other features of systemic lupus erythematosus or another systemic autoimmune rheumatic disease. Assessment of her serum anti-PLIN1 antibody levels at the time of her hospitalization for DKA and upon resolution of her disease would provide the strongest evidence for the autoimmune nature of her disease.

Nonetheless, there is a significantly increased risk of the development of autoimmune diseases within a year of delivery [5]. Pregnancy confers a unique immunological state in which hormonal changes serve to protect the semi-allogenic fetus from the maternal immune system. Driven by increased estrogen and progesterone levels, T-cell responses shift toward a Th2/regulatory state, promoting immune tolerance [6]. Hence, pregnancy has been shown to be protective in Th1-driven autoimmune conditions such as rheumatoid arthritis and multiple sclerosis. Meanwhile, prolactin promotes T-cell maturation, antigen presentation, and immunoglobulin production. The exponential rise of prolactin during delivery and breastfeeding has been blamed for the development of autoimmune flares in the postpartum period [5, 6].

Therapeutic strategies utilized in this case include highly concentrated intramuscular insulin injections in consideration of the patient’s global dearth of subcutaneous tissue for effective absorption, pioglitazone, and metformin-sitagliptin. Thiazolidinediones bind and activate PPAR-γ, thereby promoting the differentiation, maintenance, and survival of adipocytes, as well as the production of adiponectin [7]. Both thiazolidinediones and metformin have shown efficacy in improving insulin sensitivity and other metabolic parameters in patients with various lipodystrophic conditions [7, 8]. However, definitive comparison between the 2 therapies, as well as investigation of glucagon-like peptide 1 agonists and sodium glucose co-transporter 2 inhibitors, remains to be conducted in this patient population. Another potential therapy, metreleptin, a recombinant human leptin analog, is currently restricted by the Food and Drug Administration’s Risk Evaluation and Mitigation Strategy Program for patients with generalized lipodystrophy with minimal circulating leptin levels and poor response to oral antihyperglycemics and insulin therapy [9]. This is due to the observation of leptin-binding antibodies in a subset of patients and the association of metreleptin and T-cell lymphoma [10]. Given that leptin-binding antibodies show neutralizing activity in vitro, there is concern that they may diminish the therapeutic efficacy of the drug and inhibit endogenous leptin in vivo. The association between metreleptin and lymphoma may be confounded by an underlying independent association between AGL and lymphoma [11].

## Learning Points

- Administration of intramuscular insulin, thiazolidinediones, and metformin are effective pharmacological strategies in the treatment of lipodystrophy.
- Due to the strong association of AGL with autoimmunity, newly diagnosed patients should be screened for comorbid autoimmune conditions.
- Patients with AGL can undergo spontaneous remission. The development of a clinical anti-PLIN1 assay may have tremendous utility in not only identifying the etiology of AGL but also monitoring for disease remission/relapse.

## Data Availability

Original data generated and analyzed during this study are included in this published article.

## Abbreviations

AGL: acquired generalized lipodystrophy
ANA: antinuclear antibodies
ANC: absolute neutrophil count
anti-PLIN1: anti-perilipin-1; anti-U1-RNP; anti-U1-ribonucleoprotein
BMI: body mass index
DKA: diabetic ketoacidosis
HbA1c: glycated hemoglobin
PLT: platelet count
RR: reference range
WBC: white blood cell
